# Effect of different food groups on energy intake within and between individuals

**DOI:** 10.1007/s00394-022-02903-1

**Published:** 2022-05-27

**Authors:** Graham W. Horgan, Stephen Whybrow, Andrea Scalco, Tony Craig, Jennie I. Macdiarmid

**Affiliations:** 1 Biomathematics & Statistics Scotland, Aberdeen, UK; 2 Life Course and Population Health, The Rowett Institute, University of Aberdeen, Aberdeen, UK; 3 Social Economic and Geographical Sciences Research Group, The James Hutton Institute, Aberdeen, UK

**Keywords:** Food groups, Energy intake, Compensation

## Abstract

**Purpose:**

Energy intake varies day-to-day because we select different foods, and different amounts of these foods. Energy balance is not tightly regulated over the short-term, and the variability in diet results in an energy surplus or deficit. The aim of this study was to explore how consuming more, or less, than usual amounts of foods contributed towards balancing of total energy intake (TEI) within a day.

**Methods:**

Four-day food records came from 6155 adult participants of the National Diet and Nutrition Survey to study these effects. Within-individual regression models of the energy from 60 food groups on TEI were calculated. Energy intake variation within-individuals was regressed separately on the variation in amounts of each food group. Regression models were also fitted to individual four day means.

**Results:**

Within-individual coefficients ranged from about 0 for high-fibre breakfast cereals to 1.7 for sugar preserves and spreads. Three food groups (e.g. low-calorie soft drinks) tended to reduce TEI, and 13 food groups (e.g. margarine and other spreads, and alcoholic drinks) tended to elevate TEI above the energy content of the food group when more than usual amounts were consumed. Foods groups of higher energy densities, or lower fibre content (e.g. typical “snack” foods, low-fibre bread, and processed meat) tended to promote greater TEI more so than did food groups of lower energy densities (e.g. meat, fish, high-fibre foods, and potatoes).

**Conclusion:**

Different food groups vary considerably in the extent to which they affect TEI in free-living adults. The associations between consuming more, or less, than usual amounts of foods and the effects on TEI are consistent with those found in laboratory studies. Importantly, the present study found similar associations, but using a different methodology and in observational data, providing novel information on energy intake compensation.

## Introduction

Understanding the relative strengths of dietary and behavioural factors that elevate energy intake is important in understanding the development of obesity, because this could improve dietary recommendations. People vary what, and how much, they eat each day, and because there is no physiological need to match energy intake with expenditure over short time periods, this results in a day-to-day variation of energy intake around the average energy requirement.

Numerous intervention studies have demonstrated incomplete compensation for perturbed energy balance over one or a few days (e.g. 1, 2), and that the response to reductions in dietary energy is greater than the response to increases [3]. Incomplete compensation has also been seen in observational studies [4, 5].

Generally, foods that are high in fats and rapidly assimilated carbohydrate, and that are low in protein and fibre are conducive to higher levels of energy intake [6]. Non-nutritional properties, such as portion size [7], and eating rate [8] also influence energy intake.

Additionally, when the energy density (kJ/100 g) of foods is covertly increased, study participants tend to eat a similar amount of food and increase energy intake [9]. Conversely, when the energy density of foods is covertly decreased, a similar lack of change in behaviour and lower energy intake is seen. When the energy density of foods is manipulated, without greatly altering macronutrient composition or palatability, total of food intake (g) in a day varies little, resulting in higher energy intake when consuming diets of higher energy density [10]. This suggests that the weight of food eaten is more consistent than energy intake [10]. However, the amount of any particular food item or group varies a lot more, with many being zero some days as people vary their diet.

Taken together, this would suggest that the properties of foods that are associated with greater than usual energy intake, and that result in less than complete energy compensation, would lead to positive energy balances and ultimately a greater risk of body weight gain. Among the choices made when selecting what to eat on a particular day, there will be some balancing; more of one item today may be associated with less of some other items. This is partly a consequence of (or the cause of) normal and acceptable meal patterns, habits, and the combination of foods that make up those meals. The behaviour of others also has an influence [11], with more, or less, being eaten when the choices of others are seen, or more being eaten because others are present.

The effects of macronutrient composition and energy density on food and energy intake tend to be clearer in laboratory-type studies than in free-living studies, because they remove, or control for, many of the non-nutritional influences on feeding behaviour. Energy intake varies from day-to-day [6, 9] and it is valuable to consider how variability in food intake contributes to this in free-living people consuming their normal diets. The hypothesis tested in this study was that, in free-living adults who self-reported their food intake, the amount of food groups consumed over a day will differ, and those food groups will differ in how they impact total daily energy intake. To our knowledge, these compensation effects have not previously been examined in free living adults for a complete set of food groups.

## Methods

### Subjects

Self-reported dietary records were taken from the National Diet and Nutrition Survey (NDNS), 2008–2014 [12, 13]. The NDNS is a survey of the food consumption of a representative sample of people aged 1.5 years and older living in UK private households. The current analysis used diet records from adults (≥ 19 years of age, *n* = 6155).

Adults are asked to record everything that they eat and drink over four consecutive days, with amounts estimated using household measures, or weights from packaging. Participants are asked to provide recipes for composite dishes prepared at home. Food items categorised into the 60 Main Food Groups defined by the NDNS (Table 1) were used for these analyses.

**Table 1:** Food groups defined in the National Diet and Nutrition Survey, the proportion of all food items recoded were in each group and their contribution to energy and macronutrient totals

Food group	Percent of records	Percent of energy	Percent of protein	Percent of fat	Percent of CHO
1% fat milk	0.18	0.06	0.12	0.04	0.06
Artificial sweeteners	1.02	0.01	0.00	0.00	0.01
Bacon and ham	1.16	1.55	4.60	2.40	0.02
Beef veal and dishes	0.67	2.36	7.28	2.89	0.47
Beer lager cider and perry	1.13	3.17	0.62	0.00	2.01
Biscuits	1.79	3.49	1.27	3.96	4.31
Brown granary and wheatgerm bread	0.84	2.14	2.11	0.72	3.39
Buns cakes pastries and fruit pies	1.07	3.63	1.37	4.25	4.36
Burgers and kebabs	0.13	0.69	1.12	1.09	0.31
Butter	1.33	1.91	0.04	5.77	0.01
Cheese	1.46	3.07	4.82	6.83	0.06
Chicken and turkey dishes	1.18	3.37	12.40	3.37	0.49
Chips fried and roast potatoes and potato products	0.92	3.99	1.52	4.51	4.91
Chocolate confectionery	0.87	2.18	0.68	3.10	2.28
Coated chicken	0.16	0.74	1.20	1.08	0.42
Commercial toddlers foods and drinks	0.01	0.00	0.00	0.00	0.00
Crisps and savoury snacks	0.76	1.73	0.54	2.61	1.70
Dietary supplements	1.75	0.09	0.01	0.27	0.01
Eggs and egg dishes	0.99	1.96	3.59	3.87	0.21
Fruit	4.53	3.46	1.16	0.69	6.61
Fruit juice	0.94	0.91	0.27	0.05	1.85
High-fibre breakfast cereals	1.12	2.28	1.76	0.87	3.72
Ice cream	0.25	0.66	0.26	0.91	0.68
Lamb and dishes	0.17	0.72	1.87	1.11	0.10
Liver and dishes	0.06	0.14	0.30	0.29	0.00
Low fat spread	0.51	0.29	0.01	0.85	0.01
Meat pies and pastries	0.27	1.57	1.23	2.70	1.04
Miscellaneous	5.96	3.44	2.33	5.09	3.01
Nuts and seeds	0.77	1.09	0.90	2.57	0.21
Oily fish	0.35	1.13	2.71	1.99	0.07
Other bread	0.13	0.33	0.28	0.16	0.51
Other breakfast cereals	0.59	1.21	0.59	0.28	2.22
Other margarine fats and oils	1.06	0.77	0.00	2.33	0.00
Other meat and meat products	0.22	0.59	1.20	1.00	0.13
Other milk and cream	0.83	0.88	0.59	1.75	0.45
Other potatoes potato salads and dishes	1.36	2.74	1.61	0.58	4.97
Other white fish shellfish and fish dishes	0.5	0.75	3.20	0.51	0.13
Pasta rice and other cereals	2.36	7.30	5.58	4.01	11.00
Pork and dishes	0.25	0.88	2.85	1.19	0.04
Puddings	0.31	0.98	0.50	1.06	1.20
Pufa margarine and oils	0.38	0.24	0.00	0.72	0.00
Reduced fat spread	1.75	1.79	0.03	5.41	0.01
Salad and other raw vegetables	4.42	0.62	0.56	0.70	0.64
Sausages	0.41	1.65	2.31	3.06	0.56
Semi skimmed milk	7.77	2.74	5.21	2.72	2.14
Skimmed Milk	1.33	0.36	0.91	0.07	0.42
Smoothies 100% fruit and/or juice	0.02	0.04	0.01	0.01	0.07
Soft drinks low calorie	1.89	0.06	0.02	0.00	0.09
Soft drinks not low calorie	1.57	2.04	0.03	0.00	4.42
Spirits and liqueurs	0.36	0.60	0.00	0.05	0.07
Sugar confectionery	0.24	0.34	0.08	0.15	0.61
Sugars preserves and sweet spreads	5.08	2.59	0.04	0.10	5.54
Tea coffee and water	20.62	0.36	0.68	0.31	0.32
Vegetables not raw	6.77	3.42	4.52	2.52	4.04
White bread	2.53	6.91	5.75	2.24	11.38
White fish coated or fried	0.22	1.08	1.70	1.51	0.65
Whole milk	1.86	1.13	1.41	1.79	0.63
Wholemeal bread	0.98	2.26	2.41	0.82	3.46
Wine	0.94	2.13	0.08	0.00	0.31
Yogurt fromage frais and dairy desserts	0.91	1.41	1.76	1.09	1.65

For each individual we calculated the total energy intake for each day, and the energy intake from each food groups. The effect of each food group on total intake was calculated using linear regression model:

$$E=\alpha +{\beta }_{i}{x}_{i}+\varepsilon .$$

Here *E* represents the energy intake on a specific day, and *x* represents the intake from food group *i*, with $\varepsilon$ being the remaining residual variation. This represents 60 different regression models, one for each food group (*i*). The constant $\alpha$, not necessarily the same across models, includes an intercept and individual effects. To assist model fitting with the large number of individuals, *E* and ${x}_{i}$ were replaced with their deviations from individual means, i.e. they quantified how much that day’s intake, and that day’s consumption of the food group, differed from the average for that individual. Regression models were fitted by ordinary least squares. The coefficients, *β*_*i*_*,* therefore represent how much a deviation, positive or negative, from the usual intake of that item predicts deviation from the usual total energy intake.

The coefficients quantify the extent of compensation for varying intake of each food group. They can be interpreted as follows:

$$ \begin{gathered} \beta < 0\quad {\text{overcompensation}} \hfill \\ \beta = 0\quad {\text{complete compensation}} \hfill \\ \beta = 1\quad {\text{no compensation}} \hfill \\ \beta > 1\quad {\text{prompts more intake}} \hfill \\ \end{gathered} $$

In the model ${x}_{i}$, represents the energy in food group *i*, and for a few food groups (e.g. dietary supplements) this will be negligible and the will be less meaningful.

The link between varying amounts of food groups and intake between different individuals is not necessarily the same as the link between different days in the same individual, and so for comparison we repeated our regression modelling at the level of mean intakes for each individual. This involved fitting a regression model as above, but where ${x}_{i}$ represents the mean energy intake from that food group for each individual and *E* is their mean energy intake. We are now estimating the effect of more, or less, energy from each food group on a person’s total intake. The coefficients can no longer be interpreted in terms of compensation. A coefficient greater than one means that greater intake of that food group is associated with even greater total intake, which must come from associated greater intakes of other food groups also. This is to be expected as some people eat more than others. A coefficient less than one would imply that greater intake of that food group is not matched by greater intakes of other groups but is having the effect of displacing them in food choices, for example replacing sugar sweetened beverages by low-calorie alternatives.

We examined the link between the within-individual effects of each food group and that group’s energy density by estimating the energy density of each food group from the mean energy density of every record of the group in the dataset.

Basal metabolic rate (BMR) was estimated using the equations of Henry [14]. The ratio of mean reported energy intakes to estimated BMR was calculated.

## Results

Mean (SD) age of the 3618 females and 2537 males was 49.8 (± 17.6) years, height 1.67 (± 0.10) m, weight 77.4 kg (± 16.9) and BMI 27.7 (± 5.4) kg/m^2^. Mean (SD) daily energy intake was 7.4 (± 2.4) MJ, with 49.8 (± 17.6) % energy from carbohydrate, 32.6 (± 6.4) % energy from fat, 16.8% (± 3.9) energy from protein and 4.2 (± 6.6) % energy from alcohol. Mean ratio of reported energy intake to estimated BMR was 1.18 (± 0.35). A summary of participant characteristics is presented in Table 2.

**Table 2:** Characteristics of survey participants

	Female	Male	All
*n*	3618	2537	6155
Age	49.4 (17.8)	50.4 (17.3)	49.8 (17.6)
Height (cm)	158.9 (21.8)	172.1 (23.5)	164.3 (23.4)
Weight (kg)	70.4 (18.9)	83.5 (19.3)	75.7 (20.1)
BMI (kg/m^2^)	26.9 (7.3)	27.2 (6.2)	27 (6.9)
Energy (kJ)	6585 (1863)	8616 (2489)	7422 (2365)
Fat (g)	59 (22.3)	75.9 (27.6)	66 (26)
Fat % of energy	32.7 (6.4)	32.4 (6.4)	32.6 (6.4)
Protein (g)	64.3 (18.6)	82.2 (27.1)	71.7 (24.2)
Protein % of energy	17 (3.9)	16.6 (4)	16.8 (3.9)
Carbohydrate (g)	192.3 (58.9)	243.5 (75.5)	213.4 (70.9)
Carbohydrate % of energy	50 (7.9)	48.4 (8.1)	49.3 (8)

**Fig. 1:**
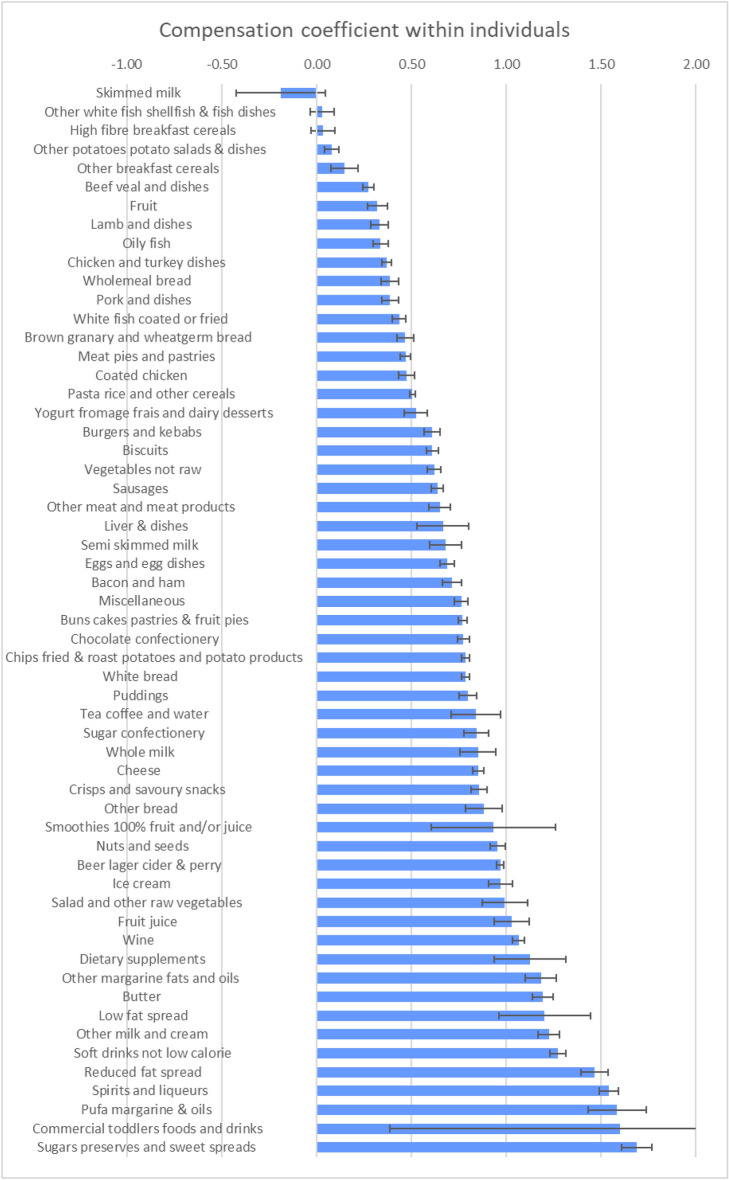
Coefficients of within-individual energy compensation for National Diet and Nutrition Survey food groups

**Fig. 2:**
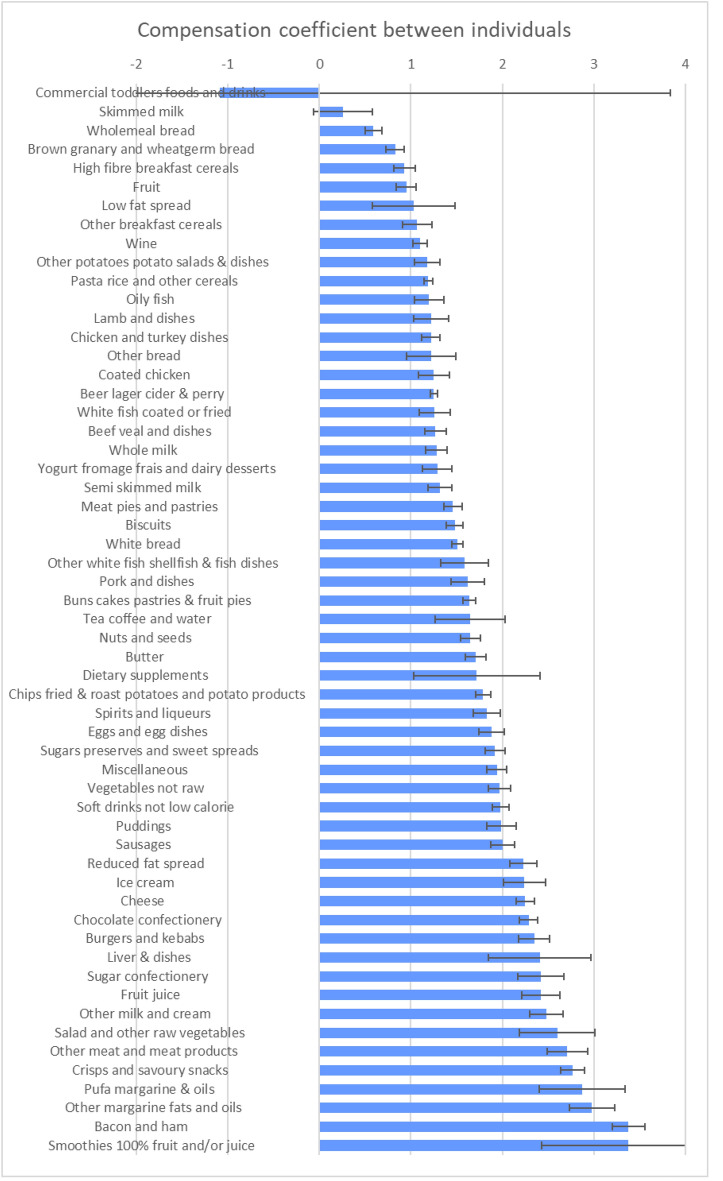
Coefficients of between-individual energy compensation for National Diet and Nutrition Survey food groups

The coefficients estimated for each food group are shown graphically in Fig. 1 (within-individual) and Fig. 2 (between-individual) and are tabulated in Table 3.

**Table 3:** Coefficients for effect of food groups on total energy intake

Food group	Beta within	SE	Beta between	SE
Artificial sweeteners	− 0.20	3.10	0.38	6.54
Bacon and ham	0.71	0.05	3.38	0.18
Beef veal and dishes	0.27	0.03	1.27	0.12
Beer lager cider and perry	0.97	0.02	1.25	0.04
Biscuits	0.61	0.03	1.48	0.09
Brown granary and wheatgerm bread	0.47	0.04	0.83	0.10
Buns cakes pastries and fruit pies	0.77	0.02	1.64	0.07
Burgers and kebabs	0.61	0.04	2.35	0.17
Butter	1.19	0.05	1.71	0.11
Cheese	0.85	0.03	2.25	0.10
Chicken and turkey dishes	0.37	0.03	1.22	0.10
Chips fried and roast potatoes and potato products	0.78	0.02	1.79	0.08
Chocolate confectionery	0.77	0.03	2.29	0.10
Coated chicken	0.47	0.04	1.25	0.17
Commercial toddlers foods and drinks	1.60	1.21	− 1.09	4.93
Crisps and savoury snacks	0.86	0.04	2.77	0.13
Dietary supplements	1.13	0.19	1.72	0.69
Eggs and egg dishes	0.69	0.04	1.88	0.14
Fruit	0.32	0.05	0.95	0.11
Fruit juice	1.03	0.09	2.42	0.21
High-fibre breakfast cereals	0.03	0.06	0.93	0.12
Ice cream	0.97	0.06	2.24	0.23
Lamb and dishes	0.33	0.05	1.22	0.19
Liver and dishes	0.67	0.14	2.41	0.56
Low fat spread	1.20	0.24	1.03	0.45
Meat pies and pastries	0.47	0.03	1.46	0.1
Miscellaneous	0.76	0.04	1.94	0.11
Nuts and seeds	0.95	0.04	1.65	0.11
Oily fish	0.34	0.04	1.20	0.16
Other bread	0.88	0.10	1.22	0.27
Other breakfast cereals	0.15	0.07	1.07	0.16
Other margarine fats and oils	1.18	0.08	2.98	0.25
Other meat and meat products	0.65	0.06	2.71	0.22
Other milk and cream	1.23	0.06	2.48	0.18
Other potatoes potato salads and dishes	0.08	0.04	1.18	0.14
Other white fish shellfish and fish dishes	0.03	0.06	1.59	0.26
Pasta rice and other cereals	0.51	0.01	1.19	0.05
Pork and dishes	0.39	0.04	1.62	0.18
Puddings	0.80	0.04	1.99	0.16
Pufa margarine and oils	1.58	0.15	2.87	0.47
Reduced fat spread	1.46	0.07	2.23	0.15
Salad and other raw vegetables	0.99	0.12	2.60	0.41
Sausages	0.64	0.03	2.00	0.13
Semi skimmed milk	0.68	0.08	1.32	0.13
Skimmed milk	− 0.19	0.24	0.26	0.32
Smoothies 100% fruit and/or juice	0.93	0.33	3.38	0.95
Soft drinks low calorie	− 1.95	0.97	5.95	2.00
Soft drinks not low calorie	1.27	0.04	1.98	0.09
Spirits and liqueurs	1.54	0.05	1.83	0.15
Sugar confectionery	0.84	0.07	2.42	0.25
Sugars preserves and sweet spreads	1.69	0.08	1.92	0.11
Tea coffee and water	0.84	0.13	1.65	0.38
Vegetables not raw	0.62	0.04	1.97	0.12
White bread	0.78	0.02	1.51	0.06
White fish coated or fried	0.43	0.04	1.26	0.17
Whole milk	0.85	0.09	1.28	0.12
Wholemeal bread	0.39	0.05	0.59	0.09
Wine	1.06	0.03	1.10	0.08
Yogurt fromage frais and dairy desserts	0.52	0.06	1.29	0.16

### Within-individual variation

For the within-individual coefficients, three food groups, soft drinks low calorie, artificial sweeteners and skimmed milk, had coefficients less than zero, suggesting that these foods led to over compensation, and lower than usual energy intakes.

Fifteen food groups had coefficients in the range of 0–0.5, suggesting that when individuals consumed more than usual amounts of these foods they partially compensated for the additional energy by up to 50%. These food groups were generally meat, fish, high-fibre foods and potatoes.

Almost half of the NDNS food groups (28 out of 59) had coefficients in the range of 0.5–1.0. The energy content of these foods was also compensated, but compensation ranged from 50% to almost zero. Many of these foods could be considered as “snack” foods (biscuits, chocolate and sugar confectionery, cakes, and savoury snacks), along with low-fibre bread, dairy products, processed meat, and vegetables.

Finally, our analysis identified 13 of the NDNS food groups that promoted further energy intake. These were spreads (e.g. margarine), including low-fat spreads, soft drinks not low calorie (i.e. sugar sweetened soft drinks), sugar preserves and the stronger alcoholic drinks (wine and spirits). The other alcoholic drinks food group (beer, lager, cider, and perry) had a coefficient just less than one (0.97) and had a similar, almost zero, compensation effect to wine. Coefficients for the three groups of alcoholic drinks were all close to, or greater than, one, indicating that these tend to promote over-consumption, and the greater the alcohol content of the drinks in these food groups, the greater the over-consumption.

### Between-individual variation

Patterns between the food groups and the between-individual coefficients were far less clear than in the within-individual coefficients. A coefficient of one in this analysis implies that greater consumption of that food group is not associated with greater consumption of other foods and so there is a simple direct effect of that greater consumption. Between individuals, almost all of the coefficients are greater than one. High-fibre foods, fruit and skimmed milk were among the few that were less than one; these are eaten in greater amounts by those who have lower than average energy intakes.

**Fig. 3:**
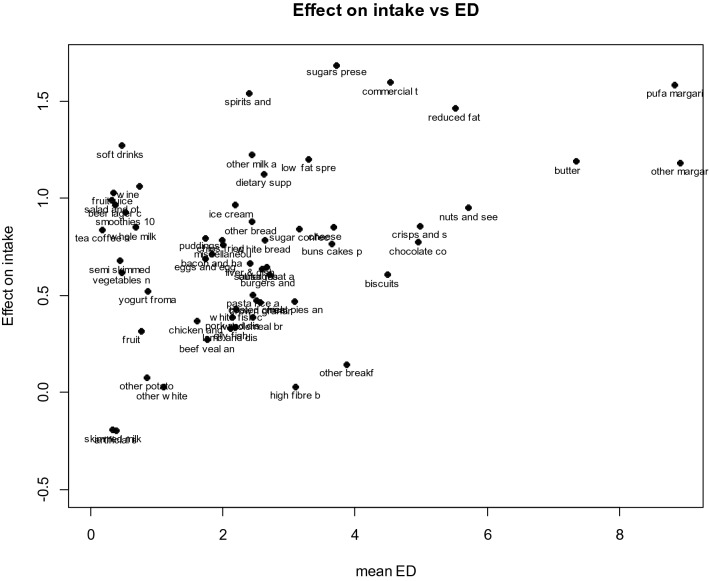
Intake coefficients versus mean energy density (ED) for NDNS food groups

Figure 3 shows within-individual coefficient against mean energy density of the food group. A positive correlation (although with some scatter) is apparent, with foods of higher energy densities tending to have higher coefficients and therefore tending to promote greater energy intakes. Breakfast cereals have much lower effects than other foods of similar energy density, while drinks, whether alcoholic, sugar sweetened soft drinks or fruit juice, tend to have higher effects.

**Fig. 4:**
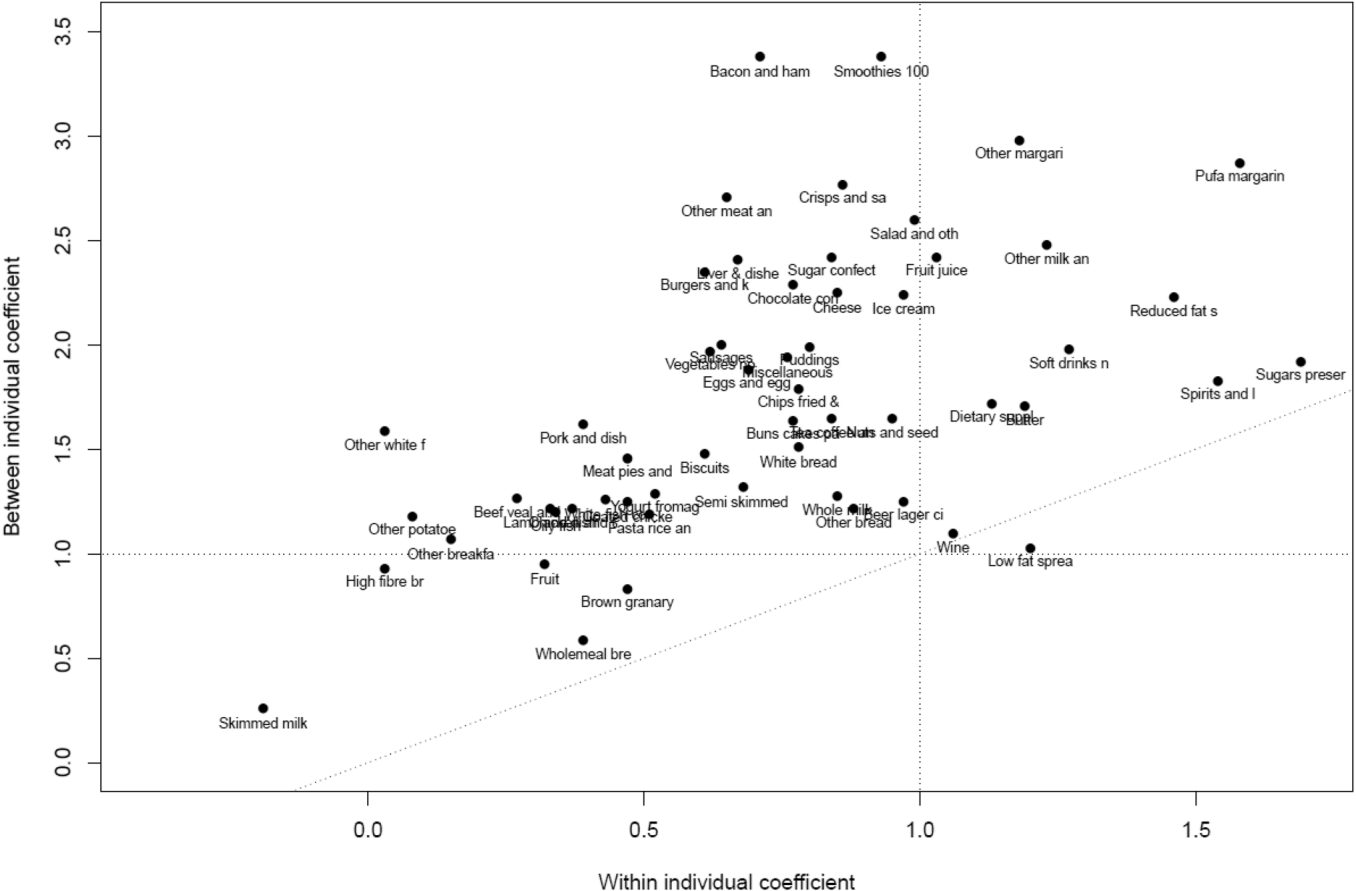
Scatter plot of between and within-individual coefficients for NDNS food groups

Figure 4 shows between-individual versus within-individual coefficients. Again, there is clearly an association, but with scatter. For example, whole milk has a similar within-individual effect as bacon and fruit smoothies, but a much lower coefficient for between-individual effects. Granary and wholemeal bread show coefficients less than one in both versions. The three alcoholic drinks food groups all have similar coefficients within-individuals and between-individuals.

## Discussion

How the properties of foods influence subsequent energy intake over the course of a day is complex, and includes non-nutritional factors as well as energy density and macronutrient composition. This study aimed to explore how different food groups contributed to differences in energy intake within days from the self-reported food intakes of adults who participated in the UK’s National Diet and Nutrition Survey.

We found that food groups vary considerably in the extent to which consuming more, or less, of them affects daily energy intake, and foods that tend to promote over-consumption under controlled laboratory-like conditions also tended to produce weaker energy intake compensatory effects in free-living people consuming their usual diets. This current study adds to the field of energy balance in that we find similar effects of foods on energy intake as reported in the literature [1, 15–17], but using a different methodology and in observational data rather than controlled intervention situations.

The variation in the compensation coefficient between the different food groups naturally leads to the question of what it is that determines their different effects on daily energy intake. One thing that is clear from Fig. 3 is that foods higher in energy density promote greater intake more than do those lower in energy density. This is also seen in laboratory studies [e.g. 18 and the energy density of overall diet is positively related to weight gain and a risk of higher BMI [19].

A tendency for foods that are higher in fibre to have lower coefficients than food groups of a similar energy density but that are higher in fat or sugar can be seen in Fig. 3. One possible effect of fibre content of foods on compensation can be seen in the within-individual coefficients of wholemeal bread (0.39), brown, granary and wheatgerm bread (0.46) and white bread (0.81). Wholemeal bread has around three times the amount of fibre as does white bread, and brown bread is around twice that of white bread. The majority of studies suggest that an increase in fibre intake reduces hunger or increases satiety, and is generally associated with a lower energy intake [20].

Compensation will, however, depend on what the bread is eaten with. Bread tends not to be eaten by itself, but typically is used to make sandwiches, and the fillings may be more energy dense than the bread.

Butter, spreads and oils also have high coefficients, and this might be expected since the also are not usually consumed by themselves. An increased intake of these therefore adds not just their energy to the daily intake, but also the energy of the foods they are added to, hence the larger coefficient.

Beta coefficients for the three groups of alcoholic drinks were all close to, or greater than, one, indicating that these tend to promote over-consumption. This is consistent with short-term pre-load/test-meal studies that clearly demonstrate that alcohol stimulates appetite to elevate food intake compared to the control (no alcohol) condition [16, 21]. Outside the laboratory setting, in moderate drinkers self-reporting their food and drink intake over several days, energy intake was higher on days when alcohol was consumed than when it was not [22]. This may be because of social facilitation, weekly variation and situation; energy intake is greater when we eat with others, at weekends [23] and when eating in restaurants [24]. Alcohol is more likely to be consumed also.

Apart from undiluted spirits, alcoholic drinks have relatively low-energy densities, and foods with low-energy densities are usually more effective at reducing hunger than highly energy dense foods [25]. The same is not always seen in drinks, as demonstrated by Haber et al. [26], who compared the effects of consuming the same weight of whole apples, pure´ed apples and apple juice on satiety. When rate of ingestion was equalised, juice was significantly less satiating than intact apples. Thus, the different form of the apples together with the removal of the fibre resulted in decreased satiety, and could potentially elevate energy intake. This is seen in the current analysis where the coefficient for fruit juice was 1.05 and fruit was 0.24, suggesting that fruit juice was less completely compensated for than was whole fruit. Furthermore, fruit smoothies with a consistency nearer to that of fruit juice than fruit, but containing all of the fibre of fruit, had a coefficient of 0.89.

The difference in within-individual coefficients between the low-calorie soft drinks (− 1.81) and the not-low-calorie soft drinks (+ 1.31) suggests that switching from sugar-sweetened to artificially sweetened drinks should promote a negative energy balance. However, the low-calorie drinks obviously have only a small amount of energy and a reduction in daily energy intake of 1.81 times this will still be a small amount of energy. Furthermore, the apparent over-compensation of low-calorie drinks may not be a cause of a lower than normal daily energy intake, but an effect of it, that is consciously trying to limit energy intake by eating less food than normal and by switching to a low-calorie drink on that day.

We were unable to include energy expenditure as a driver of energy intake in our models, because this is not measured in the NDNS. Total energy expenditure has less day-to-day variability than does energy intake [27], and there is only a loose coupling between energy expenditure and intake on a daily basis [3, 28]. Within-individual variability in energy expenditure is unlikely to be a major source of change in energy intake.

The patterns of variation in the between-individual coefficients show some similarity to those within-individuals, but also some differences. Some of the variation between individuals in their mean energy intake over four-days will be due to random within-individual variation and so affected by the same patterns. But between-individual variation is also driven by differing energy needs, which are dependent on body weight, composition and activity levels.

The results we have reported have relevance for any situations in which management of energy intake is of interest, with weight loss interventions being a prominent example. Our data are observational, and correlation and association as indicated in these observational data do not imply causation, which would require experimental work. However they do point to what natural mechanisms of balancing and compensation appear to be taking place among adults consuming their normal diet. As such they have the potential to inform any sort of dietary manipulation which is developed with intention of manipulating intake, particularly if the intention is to reduce it. If participants will have less tendency to compensate, it is plausible (but would need experimental confirmation) that it would be more effective.

## Limitations

A limitation is that there are only four-days of intake for each person; More detailed information could be obtained from longer recording periods. Being consecutive, these days are not independent, although correlations between them appeared weak (r=0.11). Four-days provides adequate data to estimate average effects in the population, though is not enough to investigate whether the patterns of compensation show heterogeneity between individuals, and so our reported effects are an aggregate across the population. Many sources of variation in food group and energy intakes, such as eating rate and energy intake rate, which are known to be associated with higher energy intake [29], were not available. Day-to-day variation in energy expenditure also was not collected, and so its role in influencing the intake variation could not be explored.

The issue of misreporting of intakes is always present, and at 1.18 the ratio of reported energy intake to BMR was lower than the minimum of 1.54 for a plausible measure of the diet over the measurement period [30]. We did not excluded probable low-energy reporters, because all participants misreport to some degree [31], and excluding only some of these could introduce bias. We did check the effect of omitting lower reporters, and found that coefficients remained similar. Misreporting will have only a small effect on the within-individual coefficients provided that misreporting is similar for each participant across their four-days, and that misreporting affects all food groups to the same level. Reported energy intake does decrease over the recording period, although at 164 kJ over four-days the effect is relatively minor [32, 33]. If a food group is under-reported more than total energy, its coefficient will be inflated by the ratio of the overall reporting percent to that of the food group.

The effects we have estimated have been aggregated across a representative sample of the UK population, but it cannot be assumed that these effects are a constant that does not vary between individuals. Considerably longer recording periods than the four-days available in the NDNS would be needed to investigate this variation.

## Conclusions

This study shows that different food groups vary considerably in the extent to which consuming them affects total daily energy intake in free-living adults. Associations between more, or less, than usual amounts of foods and the effects on total energy intake are consistent with those found in laboratory studies. Importantly, the present study found similar associations, but using a different methodology and in observational data.

Energy intake balancing through food choice is more complex and nuanced than simple effects of energy density or macronutrient composition. This study adds to the evidence that dietary recommendations based on foods, and typical combinations of foods, is likely to be more effective at limiting energy intake and combatting obesity than focussing on single macronutrients.
